# Translation and localization the Persian version of diabetes distress scale among type 2 diabetes

**DOI:** 10.1186/s13098-023-01173-z

**Published:** 2023-10-14

**Authors:** Alireza Jafari, Hadi Tehrani, Mohammadjavad Mansourian, Mahbobeh Nejatian, Mahdi Gholian‑Aval

**Affiliations:** 1 Department of Health Education and Health Promotion, School of Health, Social Development and Health Promotion Research Center, Gonabad University of Medical Sciences, Gonabad, Iran; 2 Social Determinants of Health Research Center, Mashhad University of Medical Sciences, Mashhad, Iran; 3 Department of Health Education and Health Promotion, School of Health, Mashhad University of Medical Sciences, Mashhad, Iran; 4 Student Research Committee, Gonabad University of Medical Sciences, Gonabad, Iran; 5 Social Determinants of Health Research Center, Gonabad University of Medical Sciences, Gonabad, Iran

**Keywords:** Reliability, Diabetes distress, Validity, Psychometric, Type 2 diabetes

## Abstract

**Introduction:**

The aimed of this psychometric cross-sectional research was translation and localization the Persian version of diabetes distress scale in type 2 diabetes.

**Methods:**

This psychometric cross-sectional research was translation and localization the Persian version of diabetes distress scale among 1028 type 2 diabetes in Mashhad city, Iran, 2022. Cluster sampling method was used for selection the participants. The validity and reliability of diabetes distress scale designed and evaluated by Polonsky was assessed in this study. The validity of diabetes distress scale was evaluated by face validity, content validity, and structural validity. Twenty-six type 2 diabetes were selected for evaluation the reliability of scale.

**Results:**

The factor loading of all questions of diabetes distress scale were more than 0.4 and the results of goodness-of-fit indexes showed acceptable values (for example: RMSEA = 0.076, IFI = 0.909, AGFI = 0.819, PNFI = 0.758). Cronbach’s alpha coefficient, McDonald omega coefficient and Intraclass Correlation Coefficient (ICC) showed a value of 0.950, 0.955, and 0.903, respectively for all items of diabetes distress scale. Cronbach's alpha coefficient, McDonald omega coefficient and ICC showed a value of 0.914, 0.917, and 0.893, respectively for Core Level of Distress (8 items). Also, Cronbach's alpha coefficient, McDonald omega coefficient, and ICC showed a value of 0.920, 0.928, and 0.884, respectively for all factors of Sources of Distress (21 items).

**Conclusion:**

The Persian form of diabetes distress scale with 29 items and two parts of Core Level of Distress with 8 items and Sources of Distress with 21 items and 7 factors (Hypoglycemia with 3 items, Long-term Health with 3 items, Healthcare Provider with 3 items, Interpersonal Issues with 3 items, Shame/Stigma with 3 items, Healthcare Access with 3 items, and Management Demands with 3 items) is a good scale to evaluation the status of diabetes distress in Iranian type 2 diabetes.

## Introduction

Diabetes has significant clinical concerns due to its high prevalence and its clinical relationship with disease management, drug adherence, blood sugar control and quality of life, and diabetes is associated with an increased risk of mental disorders [1, 2]. Based on the finding of study in Iran, the prevalence of type 2 diabetes in Mashhad city (Iran) was reported as 17.7% [3]. One of the problems that may face diabetes over time is diabetes distress. Diabetes distress refers to concerns and fears among people with diabetes over time because they fight with a chronic and progressive disease such as diabetes [4–6]. The results of a systematic review study showed that in type 2 diabetes patients the prevalence of diabetes distress is 36% [7]. The results of a study in Iran also showed that 48% of adults with type 2 diabetes had diabetes distress [8].

It should be noted that diabetes is not a complication with diabetes or complication caused by diabetes and only over time and with the management of diabetes may occur. Diabetes distress is distinct from clinical depression and severe depression disorder that may require separate evaluation [4–6]. Due to the stability of diabetes distress, it can cause diabetes burnout in patients if it is ignored and does not pay sufficient attention. Diabetes burnout refers to the feeling of frustration and exhaustion in the management of diabetes and may ultimately lead to the ignorance of self-care behaviors by patients [9, 10]. Ignoring self-care behaviors can have dangerous consequences and different complications for patients [11, 12].

Various studies have shown that diabetes distress and eventually diabetes burnout are important factors in predicting self-care behaviors by patients and can reduce self-care behaviors [13–15]. Therefore, it is necessary to consider the distress of diabetes and the exhaustion of diabetes in patients to prevent adverse consequences by timely diagnosis and starting treatment [13–15].

Therefore, it is necessary to examine the condition of the patients in terms of diabetes distress to prevent diabetes burnout by taking appropriate preventive programs. To check the state of diabetes distress there is need a valid tool in this field. One of the most suitable tools for examining diabetes distress designed and evaluated by Polonsky et al. [16]. This diabetes distress tool includes Core Level of Distress with 8 items and seven Sources of Distress of Hypoglycemia with 3 items, Long-term Health with 3 items, Healthcare Provider with 3 items, Interpersonal Issues with 3 items, Shame/Stigma with 3 items, Healthcare Access with 3 items, and Management Demands with 3 items[16]. Based on searches in scientific sources, the diabetes distress scale has not been studied in Iranian type 2 diabetes and the present study aimed to translate and localize the Persian version of diabetes distress scale in type 2 diabetes.

## Methods

The aimed of this psychometric cross-sectional research was translation and localization the Persian version of diabetes distress scale among type 2 diabetes in Mashhad city, Iran, 2022.

### Sample size

In this psychometric research study, the sample size of 1028 was determined for confirmatory factor analysis. For checking the factor analysis, sample size more than 1000 is excellent [17, 18].

### Sampling method

Participants were selected by cluster sampling method from health services centers (n = 5). Of the five health services centers, three centers were randomly selected. Then they referred to health services centers and the samples were selected as simple random sampling among type 2 diabetes who had the entry criteria. The data was then collected using a questionnaire and completed by the participants by self-report. Since some participants were illiterate, the information was completed by the questioner for them. Participants with inclusion criteria consists of people who had a health record in health services centers of Mashhad, have been a resident of the city of Mashhad, type 2 diabetes patients with who have passed a year since their onset of disease, and be interested in participating in the study. In this study questionnaire of people who were not completely answered were eliminated during the data analysis phase.

### Measure instrument

1. Demographic information of participants: The period of diabetes, marital status, age, occupation, sex, the age beginning the disease, and education level were surveyed.
2. Diabetes distress scale: This scale designed and evaluated by Polonsky et al. [16]. This diabetes distress scale includes Core Level of Distress with 8 items and seven Sources of Distress of Hypoglycemia with 3 items, Long-term Health with 3 items, Healthcare Provider with 3 items, Interpersonal Issues with 3 items, Shame/Stigma with 3 items, Healthcare Access with 3 items, and Management Demands with 3 items [16]. The questions are measured on a 5-choice Likert scale (Not a Problem = 1, A Slight Problem = 2, A Moderate Problem = 3, A Serious Problem = 4, A Very Serious Problem = 5) and the high score indicates higher diabetes distress in patients [16].

### Translation and cultural adoption of Persian version of scale

First, the main designer of the diabetes questionnaire was allowed to translate and psychometric the tool. Then, the cultural adaptation and translation of diabetes distress scale was done using the World Health Organization’s guideline [19] and the Persian version of the tool was prepared for evaluation the face validity, content validity, and structural validity.

### Face and content validity

To checking the quality face validity of diabetes distress scale, use of words simple, use of understandable words, and common language were evaluated. To checking the quality content validity of diabetes distress scale, grammar adoption, proper placement of each item, use of appropriate words, require time to complete scale, and importance of each item were evaluated. The quality face validity of diabetes distress scale was evaluated by two points of view of specialist group and target group. Twenty-six participants in target group surveyed the quality face validity of Persian version of diabetes distress scale. In the specialist group, nine specialists of Psychologist, Public Health, Health education and promotion surveyed the quality face validity and quality content validity of Persian version of diabetes distress scale.

### Structural validity

To checking the structural validity, the confirmatory factor analysis (CFA) was used to evaluate the components of diabetes distress scale by AMOS version 24. At first, Mahalanobis test was used to checked the outlier’s data. Then, skewness test and kurtosis test were used to checked the normality of data. To performed the CFA, the method of maximum likelihood estimation was used and CFA provided unstandardized factor loading and standardized factor loading. In this study the standardized factor loading was reported. Finally, to confirming the final mode of each scale in CFA stage, the goodness of fit indexes must have standard values. The important goodness of fit indexes that using to evaluated the final mode consist of RMSEA (root mean square error of approximation < 0.08), RMR (root mean square residual < 0.08), AGFI (adjusted goodness of fit index > 0.8), χ^2^/df (chi-square ratio to degree of freedom < 5), IFI (incremental fit index > 0.9), PGFI (parsimony goodness of fit index > 0.5), CFI (comparative fit index > 0.9), PCFI (parsimony comparative fit index > 0.5), and PNFI (parsimonious normed fit index > 0.5) [20–23].

### Reliability

Twenty-six type 2 diabetes were selected for evaluation the reliability of diabetes distress scale. The test–retest diabetes distress scale was assessed among 26 type 2 patients at two stages with a distance of 2 weeks. Then the ICC (Intraclass Correlation Coefficient) was calculated for all items of diabetes distress scale and for Core Level of Distress (with a factor and 8 items) and seven Sources of Distress (with 7 factors and 21 items). Also, internal consistency of diabetes distress scale was surveyed by Cronbach α (calculated using SPSS version 20 software) and then McDonald’s omega (calculated using JASP Version 0.11.1 software). The rate value more than 0.70 is good for internal reliability [24, 25] and rate value more than 0.80 is good for ICC [26].

## Results

### Demographic characteristics

The average (± standard deviation) age of patients was 55.17 (± 13.73). The average (± standard deviation) period of diabetes was 10.83 (± 8.38). Most of patients was female (n = 597, 58.9%), married (n = 867, 86.7%), and had elementary school (n = 294, 29.5%) (Table 1).

**Table 1:** Frequency distribution of demographic characteristics

Variables	n	%
Sex		
Male	417	41.1
Female	597	58.9
Marital status		
Married	867	86.7
Single	92	9.2
Divorced	41	4
Occupation		
Housewife	509	51.1
Employed	87	8.7
Retired	155	15.5
Self-employed	167	16.7
Laborer	53	5.3
Unemployed	26	2.6
Education level		
Illiterate	150	15
Elementary school	294	29.5
Middle school	144	14.4
High school	96	9.6
Diploma	136	13.6
Associate degree	79	7.9
Bachelor degree	80	8
Master’s degree or high degree	19	1.9

### Face and content validity

Based on the points of view of target group, five questions were modified in terms of using understandable words and use of simple words in Persian version of diabetes distress scale. Based the points of view of specialist, nine questions of Persian version of diabetes distress scale were revised in terms of using understandable words and use of appropriate words.

### CFA

**Fig. 1:**
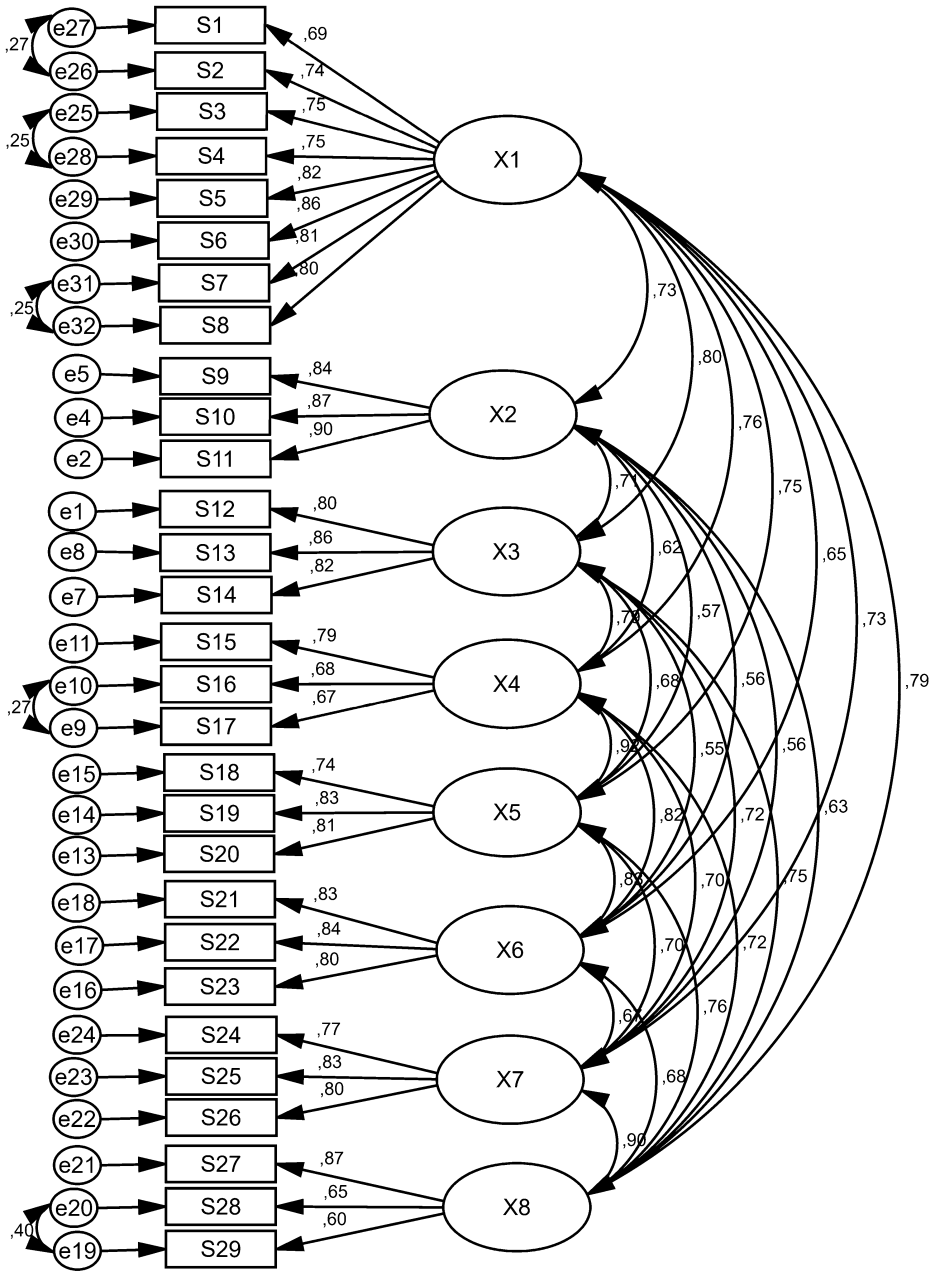
Standardized parameter estimates for the factor structure of diabetes distress scale (X1: Core Level of Distress, X2: Hypoglycemia, X3: Long-term Health, X4: Healthcare Provider, X5: Interpersonal Issues, X6: Shame/Stigma, X7: Healthcare Access, X8: Management Demands)

In CFA, the eight factors of diabetes distress scale with 29 items were surveyed. The factor loading of all questions of diabetes distress scale were more than 0.4 (Table 2) and the results of goodness-of-fit indexes showed acceptable values (for example: RMSEA = 0.076, IFI = 0.909, AGFI = 0.819, PCFI = 0.770) (Table 3). In this stage, no questions were removed and eight factors (Core Level of Distress with 8 items and seven Sources of Distress of Hypoglycemia with 3 items, Long-term Health with 3 items, Healthcare Provider with 3 items, Interpersonal Issues with 3 items, Shame/Stigma with 3 items, Healthcare Access with 3 items, and Management Demands with 3 items) with 29 items were approved (Table 2, Fig. 1).

**Table 2:** Factor loadings of the diabetes distress scale

Subscales	Items	Factor loadings (standardized regression weights)
Core level of distress	1. I feel burned out by all of the attention and effort that diabetes demands of me	0.690
	2. It bothers me that diabetes seems to control my life	0.740
	3. I am frustrated that even when I do what I am supposed to for my diabetes, it doesn’t seem to make a difference	0.747
	4. No matter how hard I try with my diabetes, it feels like it will never be good enough	0.750
	5. I am so tired of having to worry about diabetes all the time	0.818
	6. When it comes to my diabetes, I often feel like a failure	0.863
	7. It depresses me when I realize that my diabetes will likely never go away	0.813
	8. Living with diabetes is overwhelming for me	0.800
Sources of distress		
Hypoglycemia	9. I am scared that I might have a serious low glucose event when I am out in public	0.841
	10. I worry a lot that I could have a serious low glucose event	0.866
	11. I worry about having a serious low glucose event when I’m alone	0.896
Long-term Health	12. I worry a lot about developing serious complications from diabetes	0.798
	13. I can’t escape this sinking feeling that diabetes is eventually going to get me	0.862
	14. No matter what I do, I fear that serious complications from diabetes will happen to me	0.825
Healthcare Provider	15. When it comes to medical care, it upsets me that I am mostly on my own with diabetes	0.786
	16. It upsets me that I’m not really heard or understood by my healthcare provider	0.682
	17. It upsets me that my healthcare provider seems to care more about my glucose levels than about me as a person	0.671
Interpersonal Issues	18. When it comes to family and friends, it disappoints me that I am pretty much on my own with diabetes	0.737
	19. It frustrates me that people in my life tempt me to eat foods or do things that are not good for my diabetes	0.828
	20. It hurts me that many people in my life don’t understand what living with diabetes is really like	0.811
Shame/Stigma	21. It makes me feel bad that I must hide my diabetes from others	0.830
	22. It upsets me that people in my life think less of me because I have diabetes	0.843
	23. I often feel ashamed or embarrassed when other people know about my diabetes	0.802
Healthcare Access	24. I worry that I won’t be able to pay for my diabetes care, medicines or supplies	0.769
	25. I worry that I can’t get the healthy food I need for my diabetes	0.828
	26. I worry about how hard it is get to my healthcare appointments or pharmacy	0.805
Management Demands	27. It frustrates me that my eating often feels out of control	0.871
	28. I worry that I don’t pay enough attention to my diabetes	0.646
	29. It bothers me that I don’t get as much exercise as I should	0.601

**Table 3:** The model fit indicators of the diabetes distress scale

Goodness of fit indices	Confirmatory factor analysis	Acceptable value
χ^2^	2399/025	–
df	344	–
X^2^/df	6.974	< 5
P-value	0.001	P > 0.05
RMR	0.052	< 0.08
RMSEA	0.076	< 0.08
IFI	0.909	> 0.9
CFI	0.908	> 0.9
PNFI	0.758	> 0.5
PGFI	0.678	> 0.5
PCFI	0.770	> 0.5
AGFI	0.819	> 0.8

### Reliability assessment

Cronbach’s alpha coefficient showed a value of 0.950 for all items of diabetes distress scale. Also, McDonald omega coefficient and ICC showed a value of 0.955 and 0.903, respectively for all items of diabetes distress scale (29 items). Cronbach's alpha coefficient, McDonald omega coefficient, and ICC showed a value of 0.914, 0.917, and 0.893, respectively for Core Level of Distress (8 items). Also, Cronbach’s alpha coefficient, McDonald omega coefficient and ICC showed a value of 0.920, 0.928, and 0.884, respectively for all factors of Sources of Distress (21 items) (Table 4).

**Table 4:** Descriptive statistics of the diabetes distress scale

Subscales	Item	Range of score	Cronbach’s alpha coefficients	McDonald’s omega coefficients	Intraclass Correlation Coefficient (ICC)	95% Confidence Interval		P-value
						Lower Bound	Upper Bound	
Total diabetes distress scale	29	29–145	0.950	0.955	0.903	0.779	0.958	< 0.001
Core level of distress	8	8–40	0.914	0.917	0.893	0.755	0.953	< 0.001
All sources of distress (7 factors and 21 items)	21	21–105	0.920	0.928	0.884	0.731	0.950	< 0.001
Sources of distres**s**								
Hypoglycemia	3	3–15	0.938	0.949	0.847	0.648	0.934	< 0.001
Long-term Health	3	3–15	0.854	0.855	0.830	0.603	0.927	< 0.001
Healthcare Provider	3	3–15	0.673	0.745	0.767	0.467	0.898	< 0.001
Interpersonal Issues	3	3–15	0.790	0.793	0.905	0.782	0.959	< 0.001
Shame/Stigma	3	3–15	0.906	0.910	0.778	0.482	0.905	< 0.001
Healthcare Access	3	3–15	0.852	0.864	0.572	0.014	0.817	< 0.001
Management Demands	3	3–15	0.836	0.865	0.833	0.614	0.928	< 0.001

## Discussion

This study was aimed at translating, localization and validation of diabetes-related distress scale in people with type 2 diabetes in Iran. In general, this validity and reliability of this questionnaire was confirmed with 29 questions in the two main distress levels (one factor and 8 items) and distress sources (seven factors and 21 items). Distress sources include factors of Hypoglycemia (3 items), Long-term health (3 items), Health care provider (3 items), Interpersonal Issues (3 items), shame/sting (3 items), access to health care (3 items) and demand management (3 items). These factors and questions were matched with the original version of the questionnaire proposed by Polonski et al. [16].

In the CFA stage, eight factors were examined and all questionnaire questions were confirmed. The Cronbach's alpha coefficient and omega-McDonald’s coefficient and ICC coefficient were used to perform the reliability of the tool, which showed that the questionnaire had a good reliability in people with type 2 diabetes in Iran. The data of this study showed that the diabetes distress scale has an acceptable and generalizable factor structure and good internal reliability in patients with type 2 diabetes in Iran. One of the important features of this questionnaire is that it distinguishes the level and intensity of the perceived distress from its sources, which increases the accuracy of patient assessment. Also, the existence of these two distinct parts in the questionnaire in longitudinal evaluations allows to first measure the level and severity of the perceived distress. In people with high levels and severity of distress, distress resources are evaluated because in people with low distress, measurement of distress sources is not valuable.

The first part of this tool was Core Level of Distress and evaluates the level and severity of perceived distress in type 2 diabetes. This section was approved with 8 items, standard regression coefficient 0.690 to 0.863, omega McDonald coefficient 0.917, Cronbach’s alpha coefficient 0.914, and ICC 0.893. Diabetes distress is an emotional response to the burden of life with diabetes and self-care to manage diabetes [27]. Evaluation for diabetes is very important in diabetic because high perceived distress often results in worse psychiatric conditions such as depression or anxiety, etc. [28]. Fisher et al., in their study mention that the high levels of distress in patients with diabetes are significantly related to poor blood sugar control, poor self-care, low diabetes self-efficacy and poor quality of life [29].

The second part of the questionnaire was Sources of Distress and evaluates the sources of stresses and worries that patients experienced. This section was approved with 21 items and 7 factors, standard regression coefficient 0.601 to 0.896, omega McDonald coefficient 0.928, Cronbach’s alpha coefficient 0.920, and ICC 0.884. Identify the factors that cause distress helps primary care providers and doctors to provide better recommendations and decision-making about the necessary care for patients [6]. The first source of distress was “Hypoglycemia”. Hypoglycemia is one of the main concerns in managing diabetes and can prevent optimized blood sugar control and ultimately leads to various consequences such as diabetes distress [30]. Todd, and its colleague in their study showed that there is a significant relationship between awareness of hypoglycemia and diabetes distress [31]. The avoidance of hypoglycemia is crucial in managing diabetes and can be achieved through self-care behaviors such as blood glucose testing and insulin dose adjustment. It is important for patients to engage in recommended behavioral activities such as healthy eating, medication adherence, being physically active, and monitoring blood glucose levels [32].

The second source of diabetes distress was “Long-Term Health”. Factors such as emotional distress from living with diabetes, the burden of dayless management of the disease, and the perspective of its long-term complications can cause distress long-term health. So, concern about long-term health is one of the main sources of this situation. In their study, it also identified long-term health concerns as one of the most important factors in the families with patient diabetes [33]. Preventing long-term health concerns as a major source of diabetes distress requires a comprehensive approach that includes managing blood sugar levels effectively, following a healthy lifestyle, engaging in regular diabetes screening checks, and addressing social determinants of health [34].

The “Distress Healthcare Provider” and “Distress Healthcare Access” were confirmed by two other sources for diabetes distress. Lack of proper understanding of patients’ conditions, providing advice regardless of patient condition, limited counseling time, poor relationship with the physician, or problem of access to health care are the factors that can cause distress in people with diabetes [35]. Arifin et al. in a qualitative study in Indonesia, concerns of patients about health care provider were important factor in diabetes distress [36]. A study in the United States on 267 participants reported that lower diabetes distress is significantly related to higher levels of health care [35]. Health care providers should also work with patients to develop a diabetes management plan that is tailored to their individual needs and preferences and ensure that patients have access to regular diabetes screening checks [37].

Another the source for diabetes distress is “Interpersonal Issues” that confirmed in this study. This concept refers to the lack of understanding and not being supported by friends and family for self-care. Evidence suggests that interpersonal issues, for example, spouse and friends’ neglect of dietary treatment and tempting patients with forbidden foods have significant consequences for diabetes management [38].

Shame/Stigma was confirmed as another source of diabetes distress in this study. Diabetes distress can result from the social impact of diabetes such as stigma, discrimination, faced with the useful reactions of others or not understanding them by others people. People with diabetes often feel embarrassed, feel guilty, and anxiety about their condition, leading to a decrease in self-esteem and increase distress [39]. Satoshi Inagaki et al., in their study aimed at determining the prevalence of shame and stigma caused by diabetes and its relationship to psychological indicators, showed that the shame of diabetes was associated with distress among diabetes [40]. Strategies such as educating yourself and others, developing support and coping mechanisms, seeking positive role models, and working with a health care provider to develop a comprehensive diabetes management plan can be helpful in managing shame/stigma [41].

Management Demands was also confirmed as another source of diabetes distress in this study. This factor refers to one's despair of self-care behaviors such as exercise and having proper nutrition. Diabetes distress and demand management are closely linked in diabetes patients. Long-term needs to perform self-care behaviors of diabetes cause stressors that may lead to diabetes distress. Kreider in his study has identified Management Demands of diabetes patients as one of the symptoms of diabetes distress [42]. The management needs of diabetic can be challenging, but there are suggestions that can help, including building a basic knowledge of diabetes management, identifying and acknowledging your feelings, being honest with yourself, and providing care. Health noted about the challenges of diabetes management [16].

### Strengths and restrictions

One of the strengths of this study was that the research was conducted with high sample size, which can help the widespread use of this tool in future studies and research. One of the limitations of this study was that the information was completed using a questionnaire and a report in self-report way and may have some errors.

## Conclusion

The Persian form of diabetes distress scale with 29 items and two parts of Core Level of Distress (with 8 items) and Sources of Distress (with 21 items and 7 factors) was approved. Finally, this study presented a suitable scale for evaluating the state of distress in patients with diabetes in Iran, which had good credibility and reliability. This scale is the possibility of checking the level or intensity of the experienced distress and it provides the primary sources of distress separately. This will make the distress more accurately evaluate and design more appropriate interventions in this area. This feature also makes the distress level first evaluated and if the distress level is high, distress resources are evaluated. This tool can be used in both clinical and research fields to diagnose distress. However, it is suggested that future research focuses on the creation of cutting points for perceived distress to determine when a respondent should be classified as “weak”, “medium” or “high” distress.

## Data Availability

All data generated or analysed during this study are included in this published article.

## Abbreviations

X1: Core Level of Distress
X2: Hypoglycemia
X3: Long-term Health
X4: Healthcare Provider
X5: Interpersonal Issues
X6: Shame/Stigma
X7: Healthcare Access
X8: Management Demands
CFA: Confirmatory factor analysis
PCFI: Parsimony comparative fit index
AGFI: Adjusted goodness of fit index
IFI: Incremental fit index
RMR: Root mean square residual
RMSEA: Root mean square error of approximation
CFI: Comparative fit index
PNFI: Parsimonious normed fit index
χ^2^/df: Chi-square ratio to degree of freedom
PGFI: Parsimony goodness-of-fit index
